# The effects of liraglutide and metformin treatment on fracture healing in partially insulinopenic diabetic rats

**DOI:** 10.3389/fendo.2025.1703958

**Published:** 2025-10-21

**Authors:** Banu Turhan, Sönmez Sağlam, Mücahit Osman Yücel, Raşit Emin Dalaslan, Mehmet Ali Sungur, Fatih Demir, Zekeriya Okan Karaduman, Mehmet Arıcan

**Affiliations:** ^1^ Department of Pediatric Endocrinology, Atatürk Sanatoryum Education and Research Hospital, Ankara, Türkiye; ^2^ Department of Orthopedics and Traumatology, Düzce University Medical Faculty, Düzce, Türkiye; ^3^ Department of Biostatistics and Medical Informatics, Düzce University Medical Faculty, Düzce, Türkiye; ^4^ Department of Pathology, Düzce University Medical Faculty, Düzce, Türkiye

**Keywords:** diabetes mellitus, liraglutide, metformin, rat, fracture healing

## Abstract

**Purpose:**

Although Metformin has been studied, the comparative or synergistic effect with GLP-1 agonists like Liraglutide on fracture healing remains poorly characterized. This study aimed to evaluate the impact of metformin, liraglutide, and their combination on fracture healing in a rat model of partially insulinopenic diabetes mellitus (DM).

**Methods:**

Sixty male Wistar rats (10–14 weeks old, 350 ± 50g) were divided into five groups of twelve rats each: Control, DM, Met (Metformin), L (Liraglutide), and Met+L. Partially insulinopenic DM was induced in all experimental rats except the control group using streptozotocin (STZ) and nicotinamide (NA) combination. A femoral fracture was created, and a Kirschner wire was inserted retrogradely into the femoral canal. Liraglutide was injected subcutaneously at a daily dose of 0.6 mg/kg into the rats in the L and Met+L groups, and oral metformin was administered to the rats in the Met and Met+L groups daily at a dose of 180 mg/kg. On the 15^th^, 30^th^, and 45^th^ days, four rats from each group were selected randomly and euthanized, and the femurs were examined radiographically, biomechanically, and histopathologically.

**Results:**

The baseline characteristics of the rats before the study showed no significant differences between the groups (p>0.05). Biomechanical test results showed a significant main effect of group (p<0.001), indicating that overall Newton values varied across groups. Additionally, a significant main effect of experimental day was found (p<0.001), suggesting that Newton values changed across days regardless of group. Histopathological scores showed a statistically significant difference between the groups on the 15th day, with the L group having 75% scoring 7 (p=0.047), and on the 45th day, with the L and Met+L groups both having 75% scoring 9 (p=0.036). Conversely, no significant difference was found in radiological scores between the groups on the 15^th^ day (p=0.934), 30^th^ day (p=0.649), and 45^th^ day (p=0.502) of the experiment.

**Conclusion:**

Both metformin and liraglutide improve fracture healing in a partially insulinopenic diabetic rat model, and these findings suggest that liraglutide may offer a superior therapeutic advantage over metformin in accelerating fracture repair in patients with diabetes.

## 1 Introduction

Diabetes mellitus (DM), a chronic metabolic disorder, is distinguished by hyperglycemia arising from impaired insulin secretion, reduced insulin efficacy, or a combination of both factors. It ranks among the most prevalent systemic diseases globally and constitutes a significant health concern (1). By the year 2045, it is projected that approximately 800 million individuals worldwide will be affected by DM, with 90% of these cases classified as type 2 DM (2). Beyond well-documented complications such as nephropathy, neuropathy, retinopathy, and cardiovascular diseases, DM also exerts detrimental effects on bone metabolism. Notably, in patients with poor glycemic control, there is an increased risk of fractures and impaired fracture healing, which may contribute to additional morbidity and escalate treatment costs (3).

Bone healing encompasses a complex sequence of biological processes. Numerous aspects of these processes, including inflammation, angiogenesis, callus formation, and bone remodeling, may be negatively impacted in patients diagnosed with DM, owing to hyperglycemia and particular metabolic disorders observed in DM patients (4). Given that fracture healing constitutes a prolonged and challenging process, it is imperative to facilitate a smoother progression for diabetic patients to enhance their quality of life. Several clinical and experimental investigations have demonstrated that DM results in a reduction of osteoblast differentiation and activity, impaired angiogenesis, increased oxidative stress, and reduced osteoclast activity, all of which are well established to contribute to diminished bone regeneration capacity as a secondary consequence (5). Given these considerations, the pursuit of pharmacological agents that can mitigate these detrimental effects remains a significant area of scientific research.

Metformin, a first-line therapeutic agent particularly utilized in the management of type 2 DM, is administered orally. It enhances the activity of AMP-activated protein kinase (AMPK), reduces hepatic gluconeogenesis, and promotes peripheral glucose uptake. Consequently, these mechanisms significantly contribute to the regulation of blood glucose levels (6). Beyond glycemic control, prior research has demonstrated that metformin stimulates osteoblast differentiation, reduces advanced glycation end products, and facilitates improvements in bone architecture (6–8). The impact of metformin on fracture healing in patients with type 2 DM has been investigated in a limited number of studies within the existing literature, with some indicating positive effects (9). In contrast, others report no significant effect (10). Another antidiabetic medication, Liraglutide, functions as a glucagon-like peptide-1 (GLP-1) receptor agonist, and its efficacy in blood glucose regulation and protection against cardiovascular complications is well established (11). In recent years, the utilization of GLP-1 receptor agonists has increased; it has been demonstrated that in children over the age of 10 with type 2 DM, these agents can be administered either alone or in conjunction with metformin to attain effective glycemic control (12). The impact of GLP-1 receptor agonists on bone tissue has been explored in various studies, revealing increased osteoblast proliferation, reduced osteoclastogenesis, and enhanced skeletal blood flow (13–16). Nevertheless, some research indicates no beneficial effects on bone mass or fracture risk (17, 18). Current literature provides limited data regarding the influence of this drug class on fracture healing issues observed in diabetic patients.

The partially insulinopenic DM rat model created by combining streptozotocin (STZ) with nicotinamide (NA) replicates the pathophysiological features of human type 2 DM (19). By partially protecting pancreatic β-cells, NA administration along with STZ produces a state of stable moderate hyperglycemia, reflecting the partial insulin deficiency seen in type 2 DM. Utilization of this model enables the exploration of the mechanisms underlying diabetic fracture healing and facilitates the assessment of potential therapeutic interventions.

Although issues related to DM-associated fracture healing are well documented, there exists a paucity of experimental research examining the impact of certain widely used antidiabetic medications on this process. While some investigations have explored the effects of metformin and liraglutide on bone metabolism, there are currently no studies in the literature that compare the effects of these two drugs or examine the outcomes of combined therapy. It is hypothesized that treatment with metformin and liraglutide, both individually and in combination, enhances fracture healing in rats with diabetes, potentially restoring outcomes to levels comparable to those of non-diabetic controls. This study aims to evaluate the impact of metformin, liraglutide, and their combination on fracture healing in a rat model of partially insulinopenic DM, to provide insights into potential translational applications in clinical practice.

## 2 Materials and methods

### 2.1 Animals

The study was conducted in accordance with the guidelines of the Helsinki Declaration and received approval from the Düzce University Animal Experiments Local Ethics Committee (protocol code 2024/03/02, approval date: March 20, 2024). The male Wistar rats (n = 60) utilized in this research were sourced from the Düzce University Animal Research and Application Center. These rats were approximately 10 to 14 weeks old, with an average weight of 350 ± 30 grams, and they were observed for any health concerns during a 15-day adaptation period. They were housed in polycarbonate cages within a temperature-controlled environment (22–24 °C) under a 12-hour light/dark cycle. Standard pellets and water were supplied ad libitum.

Twelve rats were selected through simple randomization and allocated to the control group, and during this process, the researchers remained blinded. The remaining forty-eight rats were induced with a partially insulinopenic DM model via administration of a combination of STZ and NA (20).

### 2.2 Diabetes induction

At the beginning of the study, the blood glucose levels, weights, and body lengths (from the nose to the anus and from the nose to the tail end) of all the rats were measured and recorded. Partially insulinopenic DM was induced in rats (n=48) in diabetic groups that were fasted overnight (19).

NA(Sigma-Aldrich) was dissolved in a 0.9% sodium chloride solution and adjusted to a concentration of 230 mg/ml. It was then administered intraperitoneally (i.p.) at a dose of 230 mg/kg. Fifteen minutes after applying NA, STZ (Glentham, England), prepared in citrate buffer solution (0.1 M, pH 4.5) immediately before use, was given at a dose of 65 mg/kg (i.p.) to each rat. After the injections, the animals, housed in cages with six animals per cage, had unlimited access to standard feed and drinking water. One week later, blood samples were collected from the tail vein and analyzed for fasting glucose levels using a glucometer (Accu-Check, Roche). Rats exhibiting blood glucose levels exceeding approximately 250 mg/dL were classified as diabetic and subsequently selected for further experiments (21). Rats with blood glucose levels below this threshold were initially planned to be excluded from the study; however, evaluations showed that all rats given STZ/NA had blood glucose levels above the threshold. The diabetic rats were subsequently allocated to respective treatment groups through simple randomization, with the researchers maintaining blindness throughout this process.

Control (n=12): Non-diabetic group, not taking any medication.

DM (n=12): Diabetic group, not taking any medication.

Met (n=12): Diabetic group, on oral metformin.

L (n=12): Diabetic group, receiving liraglutide subcutaneously (s.c.).

Met+L (n=12): Diabetic group, taking both oral metformin and liraglutide (s.c.).

The blood glucose levels and weights of the rats were measured and recorded on a weekly basis. Liraglutide (Novo Nordisk) was injected at a daily dose of 0.6 mg/kg (s.c.) into the rats in the L and Met+L groups (22); and metformin (Sigma-Aldrich), dissolved in sterile distilled water, was administered by gavage to the rats in the Met and Met+L groups daily at a dose of 180 mg/kg (23) throughout the experimental period.

### 2.3 Surgical technique

All rats were weighed using a sensitive electronic scale, and the necessary anesthetic dose was adjusted accordingly. A combination of 50 mg/kg ketamine (Eczacıbaşı, Turkey) and 10 mg/kg xylazine (Bioveta, Turkey) was administered via the left groin (i.p.). After testing the effectiveness of anesthesia, the right knee and thigh areas of the rats were shaved and disinfected with povidone-iodine (Batticon, Adeka, Turkey). A right femoral fracture was created using the Einhorn closed fracture model (22). After fracture creation, a 1 cm incision was made at the anterior knee. The medial parapatellar approach was used to open the capsule, and the patella was laterally displaced to expose the femoral condyles. For fracture stabilization, a 0.45-mm Kirschner wire was inserted retrogradely into the femoral canal using an electric motor. The excess wire was cut at the level of the condyles and embedded into the medullary canal to prevent skin irritation. The incision was closed, and an X-ray was taken to confirm the fracture. Post-surgery, rats received fentanyl citrate (Polifarma, Türkiye) at 0.02 mg/kg (s.c.) for three days to manage pain. A veterinarian specialist monitored the rats, with six animals housed per cage. Prophylactic antibiotic treatment was not given before or after surgery to avoid affecting the fracture healing process.

On the 15^th^, 30^th^, and 45^th^ days, four rats from each group were selected randomly and euthanized. An intraperitoneal overdose of sodium pentobarbital (Narcoren-Rhone Merieux) at a dose of 150 mg/kg was administered to the rats. The animals’ death was confirmed through intracardiac puncture (24). After euthanasia, the right femur bones of the rats were dissected and disarticulated from the hip and knee joints.

The soft tissues enveloping the femur were meticulously removed from the bone without inflicting damage upon the callus tissue. Since the callus tissues that developed within 15 days do not show adequate signs of fusion and lack the strength needed for biomechanical testing, the femurs collected on day 15 were only examined using radiology and histopathology. The femurs collected at 30 and 45 days were evaluated through radiographic, biomechanical, and histopathological analyses.

### 2.4 Radiological evaluation

Anteroposterior femur radiographs were taken of all femurs from the sacrificed rats on days 15, 30, and 45 after removal of the Kirschner wire. Scoring was performed according to the Lane and Sandhu grading system (25) by three independent orthopedic surgeons who were not involved in this study.

### 2.5 Biomechanical evaluation

After radiological evaluation, the femurs underwent biomechanical testing on the same day of sacrifice. Analyses were performed using the BMT-E series material testing machine (Besmak, Türkiye) at the Düzce University Application Center of Scientific and Technological Research. A three-point bending test was conducted to assess the biomechanical properties of fracture healing at days 30 and 45. The femur was placed on two loading bars, with an 18 mm distance between them. The movable head of the testing machine applied pressure to the center of the callus at a rate of 2 mm/min until the bone fractured. The highest force in Newtons (N) just before the fracture for each specimen was recorded.

### 2.6 Histopathological evaluation

At the conclusion of the biomechanical assessment, the specimens were preserved in 10% neutral-buffered formalin. Subsequently, the samples underwent decalcification in a 10% formic acid solution at room temperature over a period of two weeks. Following verification of complete decalcification, the tissues were subjected to routine processing and embedded in paraffin blocks.

**Figure 1:**
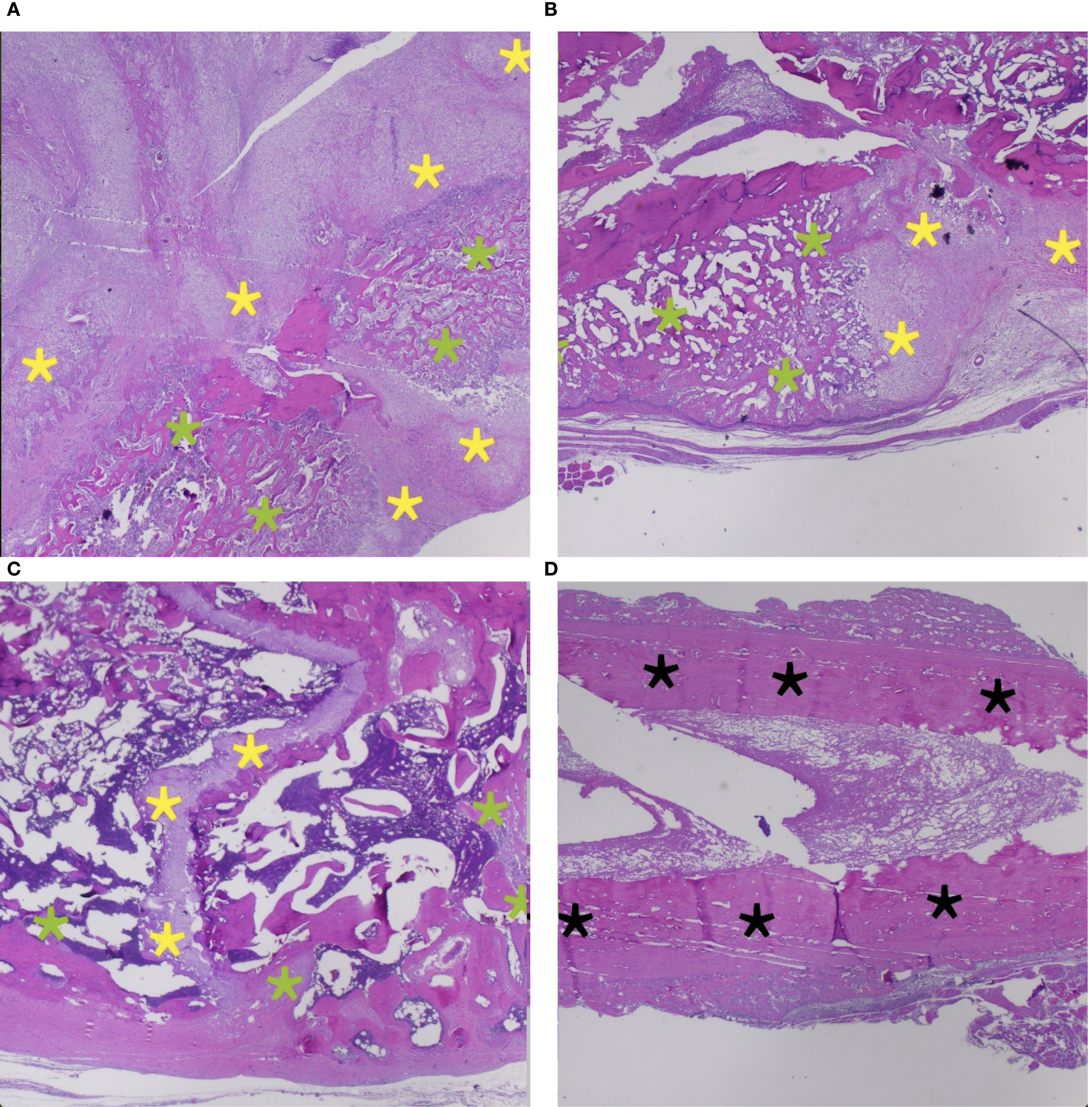
Representative histological images depicting various stages of fracture healing (H&E staining). **(A)** Predominantly cartilage-rich callus formation (yellow asterisks) with minimal immature bone component (green asterisks), consistent with Score 6. **(B)** A mixture of immature bone (green asterisks) and cartilage (yellow asterisks), indicating active endochondral ossification — consistent with Score 7. **(C)** Extensive immature bone formation bridging the fracture site (green asterisks) with minimal residual cartilage (yellow asterisks), consistent with Score 8. **(D)** Dense mature lamellar bone (black asterisks) fully bridging the fracture site, signifying advanced healing — consistent with Score 10.

Sections of 4 μm thickness were prepared from each block and stained with hematoxylin and eosin (H&E) for histological analysis. Fracture healing was assessed using the histologic scoring system described by Huo et al. (26). This numerical score, ranging from 1 to 10, is based on the dominant tissue type present at the fracture site, including fibrous tissue, cartilage, immature bone, and mature bone. All histologic evaluations were conducted under light microscopy by two independent, blinded pathologists, and average scores were used for statistical analysis. Representative histologic images from various stages of fracture healing are shown in **Figure 1**.

### 2.7 Statistical analysis

IBM SPSS Statistics v.22 (IBM Corp., 2013, Armonk, NY, USA) was used as the statistical package for analysis. Before conducting inferential tests, normality was checked using the Shapiro-Wilk test, and skewness and kurtosis values were examined. Homogeneity of variances across groups was assessed with Levene’s test. Differences between groups were analyzed using one-way ANOVA, followed by an LSD *post hoc* test for pairwise comparisons. For the within-subject design with repeated measurements, repeated measures ANOVA was used to analyze changes over time and potential time × group interactions. Bonferroni and LSD tests were used for within-group differences and multiple comparisons when necessary. Because of an anticipated reduction in sample size over time, a linear mixed-effects model (LMM) was employed to analyze repeated data, accounting for within-subject correlation and unbalanced data due to scheduled animal sacrifices at different time points. The model was estimated using the Restricted Maximum Likelihood (REML) method. The Fisher-Freeman-Halton test, with a Bonferroni-adjusted Z-test for comparing column proportions, was used to analyze categorical data. A p-value of less than 0.05 was considered statistically significant in all analyses.

## 3 Results

The baseline characteristics of the rats before the study show no significant differences between the groups; all rats had similar nose to anus and nose to tail end lengths (p=0.998 and 0.970, respectively), weights (p=0.999), and blood glucose levels (p=0.857). The mean values of these measurements prior to the study are provided in **Table 1**.

**Table 1:** Distribution of animals’ height (cm), weight (g), and blood glucose levels (mg/dl) before the study.

Parameter	Control	DM	Met	L	Met+L	p
Length-nose to anus (cm)	20.75 ± 1.42	20.92 ± 1.68	20.75 ± 1.22	20.75 ± 1.42	20.83 ± 1.34	0.998
Length-nose to tail end (cm)	41.00 ± 2.13	40.58 ± 1.98	40.50 ± 2.20	40.83 ± 2.37	40.50 ± 1.93	0.970
Weight (g)	379.33 ± 8.60	379.58 ± 9.15	379.25 ± 5.99	379.08 ± 8.37	379.50 ± 7.98	0.999
Initial blood glucose (mg/dl)	100.42 ± 9.28	98.75 ± 8.56	100.33 ± 9.58	96.75 ± 11.34	98.25 ± 6.99	0.857

DM, Diabetes mellitus; Met, Metformin; L, Liraglutide; cm, centimeters; mg, milligrams; dl, deciliters; g, grams.

Analysis of blood glucose levels before and during treatment revealed significant differences between the control and diabetic groups induced by STZ (p < 0.001). All four DM-induced groups exhibited significant increases after STZ injection, as indicated by the comparison of pre- and post-injection values (p<0.001 for all within-group comparisons). However, there were no significant differences between the different experimental weeks of treatment within these groups (p>0.05 for all within-group comparisons of weeks 1 to 6). Additionally, the control group, which did not receive STZ, exhibited no significant changes in blood glucose levels from the beginning to the end of the experimental period (p>0.05 for all within-group comparisons) (**Table 2**).

**Table 2:** Comparison of blood glucose levels (mg/dl) between groups during the 6-week study period.

Time point	Control	DM	Met	L	Met+L	p
Baseline	100.42 ± 9.28	98.75 ± 8.56	100.33 ± 9.58	96.75 ± 11.34	98.25 ± 6.99	0.857
STZ	98.50 ± 6.42^a^	345.42 ± 45.05^b^	347.08 ± 44.03^b^	355.33 ± 32.26^b^	343.42 ± 31.43^b^	**<0.001**
Week 1	97.42 ± 5.65^a^	363.33 ± 23.97^e^	280.75 ± 31.81^d^	244.25 ± 31.16^c^	208.42 ± 10.16^b^	**<0.001**
Week 2	99.33 ± 10.80^a^	368.67 ± 22.88^e^	276.17 ± 32.52^d^	231.00 ± 28.96^c^	199.17 ± 14.15^b^	**<0.001**
Week 3	95.88 ± 9.95^a^	369.13 ± 32.38^e^	270.00 ± 22.44^d^	231.75 ± 15.52^c^	190.63 ± 6.63^b^	**<0.001**
Week 4	92.88 ± 8.03^a^	378.38 ± 32.14^e^	265.75 ± 15.74^d^	222.50 ± 29.23^c^	191.25 ± 5.34^b^	**<0.001**
Week 5	98.75 ± 8.69^a^	365.00 ± 28.37^d^	266.50 ± 9.29^c^	222.00 ± 10.00^b^	188.25 ± 12.95^b^	**<0.001**
Week 6	100.75 ± 3.10^a^	369.00 ± 18.89^e^	270.25 ± 16.96^d^	219.75 ± 13.20^c^	189.50 ± 7.72^b^	**<0.001**

STZ, 1 week after streptozotocin application; DM, Diabetes mellitus; Met, Metformin; L, Liraglutide; mg, milligrams; dl, deciliters; ^a,b,c,d,e^, different superscript letters denote significant differences between the groups in each measurement time according to the post hoc test, with-in group pairwise comparisons showed no significant changes across the study period in the control group (p>0.05 for all), all STZ-induced groups exhibited significant increases from baseline to post-STZ and subsequent weeks (p<0.001 for all), with no significant differences between weeks 1 to 6 (p>0.05 for all within-group comparisons of weeks 1 to 6). Due to the extensive number of comparisons, detailed p-values are provided in the Supplementary **Table 1**.

Bold values indicate statistically significant differences (p < 0.05).

Throughout the study, the diabetic groups consistently exhibited lower weights compared to the control group, from the third week onward until the experiment’s conclusion (p = 0.038, p = 0.003, p = 0.003, and p < 0.001, respectively). In the control group, body weight decreased during the first and second weeks of treatment, followed by an increase in the third week (p<0.001). Similarly, in the four diabetic groups, weight initially decreased; subsequently, it increased in the third week; however, these weights remained significantly lower than those observed in the control group (p<0.001) (**Table 3**).

**Table 3:** Comparison of weights (g) between groups during the 6-week study period.

Time point	Control	DM	Met	L	Met+L	p
Baseline	379.33 ± 8.60	379.58 ± 9.15	379.25 ± 5.99	379.08 ± 8.37	379.50 ± 7.98	0.999
Week 1	356.05 ± 12.83	358.17 ± 11.95	358.33 ± 5.88	361.75 ± 12.66	361.92 ± 12.08	0.673
Week 2	349.92 ± 12.18	344.92 ± 11.29	349.83 ± 6.69	352.08 ± 12.80	355.25 ± 12.63	0.271
Week 3	362.25 ± 12.20^b^	347.38 ± 10.41^a^	355.38 ± 4.27^ab^	359.25 ± 11.76^b^	361.75 ± 10.63^b^	**0.038**
Week 4	370.13 ± 11.14^b^	351.63 ± 8.40^a^	362.38 ± 4.27^b^	365.25 ± 10.54^b^	369.00 ± 10.70^b^	**0.003**
Week 5	381.75 ± 9.07^c^	352.75 ± 9.74^a^	368.25 ± 2.99^b^	373.00 ± 8.04^bc^	376.25 ± 10.90^bc^	**0.003**
Week 6	400.75 ± 5.44^c^	353.50 ± 8.39^a^	377.75 ± 7.27^b^	381.50 ± 7.77^b^	380.25 ± 13.93^b^	**<0.001**

DM, Diabetes mellitus; Met, Metformin; L, Liraglutide; g, Grams. ^a,b,c,d,e^, different superscript letters denote significant differences between the groups in each measurement time according to the post hoc test, within-group pairwise comparisons showed complex patterns of significant and non-significant differences across groups and time points. Due to the extensive number of comparisons, detailed p-values are presented in the **Supplementary Table 2**.

Bold values indicate statistically significant differences (p < 0.05).

When evaluating the biomechanical test results, no significant interaction was found between group and experimental day (p=0.525), indicating that the effect of the group did not depend on the experimental day. However, there was a significant main effect of group (p < 0.001), indicating that overall Newton values differed across groups; on the 30th day, the Newton scores of the control and Met+L groups were similar, and by the 45th day, the Met and L groups showed comparable scores. Additionally, a significant main effect of experimental day was found (p<0.001), suggesting that Newton values changed across days regardless of group. Although no significant interaction was observed, exploratory *post hoc* analyses, adjusted for multiple testing using the Bonferroni correction, were performed within each group to investigate further specific differences between groups and days (**Table 4**).

**Table 4:** Comparison of biomechanical evaluation (Newton values) across the groups by experimental day.

Experimental day	Control	DM	Met	L	Met+L	p_b_
Newton-30^th^ day	101.35 ± 18.64^a^	38.85 ± 5.53^c^	51.33 ± 18.28^c^	75.70 ± 4.81^b^	93.50 ± 7.31^a^	**<0.001**
Newton-45^th^ day	124.05 ± 6.02^a^	53.50 ± 9.78^d^	78.28 ± 4.24^c^	89.90 ± 8.40^c^	105.13 ± 4.82^b^	**<0.001**
p_w_	**0.004**	**0.050**	**0.001**	0.057	0.116	0.525

DM, Diabetes mellitus; Met, Metformin; L, Liraglutide; p_b,_ between groups; p_w,_ within groups, ^a,b,c,d,e^, different superscript letters denote significant differences between the groups in each measurement time according to the post hoc test.

Bold values indicate statistically significant differences (p < 0.05).

When the overall histopathological scores were analyzed in detail, a statistically significant difference between the groups was observed on the 15^th^ day (p=0.047) and 45^th^ day (p=0.036), but not on the 30^th^ day (p=0.128). The score of 7 was observed in 75% of the L group on the 15th day, and the score of 9 was observed in 75% of both the L and Met+L groups on the 45th day. Conversely, no significant difference was found in radiological scores between the groups on the 15^th^ day (p=0.934), 30^th^ day (p=0.649), and 45^th^ day (p=0.502) of the experiment (**Table 5**).

**Table 5:** Comparisons of histopathological and radiological evaluations across groups by experimental day.

Evaluation parameter	Control	DM	Met	L	Met+L	p
HS - day15, n (%)						
5	0 (0.0)^a^	3 (75.0)^b^	2 (50.0)^ab^	0 (0.0)^a^	0 (0.0)^a^	**0.047**
6	2 (50.0)	1 (25.0)	2 (50.0)	1 (25.0)	3 (75.0)	
7	2 (50.0)^ab^	0 (0.0)^a^	0 (0.0)^a^	3 (75.0)^b^	1 (25.0)^a^	
HS - day30, n (%)						
6	0 (0.0)	0 (0.0)	1 (25.0)	0 (0.0)	0 (0.0)	0.128
7	0 (0.0)	3 (75.0)	3 (75.0)	2 (50.0)	1 (25.0)	
8	3 (75.0)	1 (25.0)	0 (0.0)	2 (50.0)	1 (25.0)	
9	1 (25.0)	0 (0.0)	0 (0.0)	0 (0.0)	2 (50.0)	
HS - day45, n (%)						
7	0 (0.0)	1 (25.0)	1 (25.0)	0 (0.0)	0 (0.0)	**0.036**
8	1 (25.0)^ab^	3 (75.0)^b^	1 (25.0)^ab^	1 (25.0)^ab^	0 (0.0)^a^	
9	0 (0.0)^a^	0 (0.0)^a^	2 (50.0)^ab^	3 (75.0)^b^	3 (75.0)^b^	
10	3 (75.0)^b^	0 (0.0)^a^	0 (0.0)^a^	0 (0.0)^a^	1 (25.0)^ab^	
RS - day15, n (%)						
0	1 (25.0)	1 (25.0)	1 (25.0)	0 (0.0)	1 (25.0)	0.934
1	2 (50.0)	3 (75.0)	3 (75.0)	4 (100)	3 (75.0)	
2	1 (25.0)	0 (0.0)	0 (0.0)	0 (0.0)	0 (0.0)	
RS - day30, n (%)						
0	0 (0.0)	1 (25.0)	0 (0.0)	0 (0.0)	0 (0.0)	0.649
1	0 (0.0)	2 (50.0)	2 (50.0)	1 (25.0)	0 (0.0)	
2	2 (50.0)	1 (25.0)	1 (25.0)	2 (50.0)	3 (75.0)	
3	2 (50.0)	0 (0.0)	1 (25.0)	1 (25.0)	1 (25.0)	
RS - day45, n (%)						
1	0 (0.0)	1 (25.0)	0 (0.0)	0 (0.0)	0 (0.0)	0.502
2	0 (0.0)	2 (50.0)	2 (50.0)	1 (25.0)	0 (0.0)	
3	2 (50.0)	1 (25.0)	1 (25.0)	2 (50.0)	1 (25.0)	
4	2 (50.0)	0 (0.0)	1 (25.0)	1 (25.0)	3 (75.0)	

HS, Histopathological score; RS, Radiological score; DM, Diabetes mellitus; Met, Metformin; L, Liraglutide; p_b_, between groups; p_w_, within groups; ^a,b,c,d,e^, different superscript letters denote significant differences between the groups in each measurement time according to the post hoc test.

Bold values indicate statistically significant differences (p < 0.05).

## 4 Discussion

This study examined the effects of metformin, liraglutide, and their combination on fracture healing through radiological, biomechanical, and histopathological methods in an experimental partially insulinopenic DM rat model created with the STZ-NA combination. The data showed that DM significantly impairs fracture healing and that metformin and liraglutide, particularly when used together, may improve biomechanical and histopathological fracture healing outcomes.

Fracture healing involves stages such as inflammation, repair, and remodeling. After a fracture, an inflammatory response occurs first, with activated immune cells interacting with bone cells. This is followed by the repair phase, where bone bridges form, and finally the remodeling of the resulting callus tissue (27). Many stages of fracture healing are significantly impacted in DM and present clinically as issues like delayed union and nonunion. It has been shown that inflammation, angiogenesis, endochondral ossification, and remodeling are affected, leading to serious fracture healing problems in patients with type 2 DM (28). When examining the biological causes of impaired fracture healing, it is observed that oxidative stress and chronic inflammation—caused by factors such as hyperglycemia and advanced glycation end products (AGEs) in diabetic patients—disrupt osteoblast differentiation and reduce osteoclast activity, causing impaired bone remodeling (29). Recent studies have shown that in patients with type 2 DM, impairments in fracture healing occur due to defects in mesenchymal and skeletal system progenitor cell functions and ciliary signaling pathways (30, 31). Furthermore, some studies indicate that reduced antioxidant defense systems associated with oxidative stress in type 2 DM patients are a significant factor impairing fracture healing (32). In our study, fracture healing scores were worse in the group with induced DM and no antidiabetic treatment compared to the other groups, as assessed biomechanically and histopathologically.

Metformin is the first-line oral medication used to treat patients with type 2 DM (33). It has been shown that metformin activates the AMPK complex, thereby improving glycemic control, enhancing osteoblastic differentiation, reducing AGE, and supporting angiogenesis (34, 35). Some experimental studies indicate that metformin promotes the formation of type H vessels in animal models of type 2 DM, accelerates endochondral ossification, and aids fracture healing (7, 8, 36). Additionally, it has been demonstrated to speed up the healing of bone defects in a type 2 DM rat model by suppressing neutrophil extracellular traps (NETs) observed around these defects (37). Despite these positive effects, some studies have shown that metformin has no impact on fracture healing (10) and may even exert negative effects (9). In our study, diabetic rats treated with metformin achieved better biomechanical and histopathological fracture healing scores compared to untreated diabetic rats. These findings support previous research indicating that metformin has beneficial effects on fracture healing in a partially insulinopenic DM rat model.

GLP-1 is secreted by L-cells, which are intestinal epithelial endocrine cells, in response to food entering the intestinal lumen. By binding to GLP-1 receptors on pancreatic beta cells, it promotes glucose-dependent insulin secretion by pancreatic islets (38). It has been shown that GLP-1 secretion and activation are decreased in patients with type 2 DM, and that significant increases in insulin levels occur when GLP-1 infusion above normal levels is given to these patients (39). However, because natural GLP-1 is rapidly inactivated by dipeptidyl peptidase-4 (DPP-4), using GLP-1 receptor agonists (GLP-1Ra) is more effective (40). Some experimental studies have indicated that deletion of the GLP-1 receptor leads to increased osteopenia and changes in collagen within the bone matrix (41, 42). Additionally, administering GLP-1Ra for three days has been shown to raise trabecular bone mass and osteoblast markers in diabetic and non-diabetic rats (43). In an experimental osteoporotic fracture model, the GLP-1Ra liraglutide was reported to enhance callus formation and positively influence remodeling (44).

On a molecular level, the beneficial effects of liraglutide may be linked to the activation of the cAMP-dependent Protein Kinase A (cAMP/PKA) signaling cascade following GLP-1 receptor stimulation. This pathway has been demonstrated to enhance osteoblast differentiation, increase collagen synthesis, and suppress osteoclast activity, thereby facilitating callus formation and remodeling (45). Concurrently, metformin exerts its effects primarily by activating AMPK, which improves mitochondrial function, reduces oxidative stress, and promotes angiogenesis. AMPK signaling has also been reported to stimulate osteogenic differentiation of mesenchymal stem cells and inhibit osteoclastogenesis (37). These complementary mechanisms may elucidate why the combination of liraglutide and metformin yielded superior biomechanical and histopathological outcomes in our study.

In addition to all the experimental studies conducted, some clinical studies and meta-analyses have also demonstrated that Liraglutide increases bone mineral density, reduces bone resorption, and accelerates bone formation, thereby improving fracture healing and reducing bone loss observed in patients with osteoporosis (3, 13). Some studies have also reported that, aside from these positive effects, GLP-1Ra drugs have no modifying impact on bone metabolism in type 2 DM patients, and no superiority over other antidiabetic drugs has been found (39, 45). A limited number of previous studies reported that liraglutide, either alone or combined with insulin, had positive effects on fracture healing in type 2 DM rats (46). In our study, it was observed that blood glucose control remained stable and balanced in diabetic rats treated with liraglutide, and that better results were obtained in the biomechanical and histopathological scores of fracture healing compared to the diabetic control group and the diabetic group treated with metformin.

When analyzing the results of our study, the most notable finding is that the biomechanical and histopathological healing scores in the diabetic group receiving combination therapy with metformin and liraglutide were significantly better than those in all other diabetic groups. In addition, healing scores like those of the non-diabetic control group were achieved with this combination therapy. A similar study reported that the combination of liraglutide and insulin had more effective results on fracture healing in diabetic rats than the same treatments given alone (46). The superior effects of the combination therapy may partly result from more stable blood glucose levels compared to liraglutide or metformin alone, which can influence bone cells and fracture healing. A randomized controlled prospective clinical study showed that liraglutide alone or combined with metformin led to significant improvements in blood glucose levels in children and adolescents aged 10 years and older with type 2 DM, and that it can be used in a safe manner in this age group (12).

Biomechanical analysis showed significant differences between groups regardless of the day. On both day 30 and day 45, the control group achieved the highest scores, closely matching the results of the Met+L group, while the DM group had the lowest scores at both measurement times. Previous studies in diabetic rats and humans have also indicated that biomechanical strength is reduced in DM compared to normal bone (9, 47). A study examining the effects of liraglutide on fracture healing in an osteoporotic rat model found that rats treated with liraglutide experienced better biomechanical outcomes (48). When radiological fracture healing scores were assessed, although the control and Met+L groups had higher scores than the other groups at Day 45, this difference was not statistically significant. The sensitivity of evaluating callus maturation and fracture healing with radiography is limited, and methods such as micro-CT may be necessary in studies examining early and mid-term fracture healing, as in the present study (46, 49). Histopathological examination is also one of the most effective and reliable methods for evaluating fracture healing (50). When analyzing our study data, no significant differences were found between the groups on day 30, while on days 15 and 45, the control and Met+L groups showed the best healing scores, and the DM group had the worst scores during both time points. These findings align with the literature and support the positive effects of combination therapy with metformin and liraglutide on fracture healing (44, 46, 51).

One of the strengths of our study is that the partially insulinopenic DM rat model created with the STZ-NA combination resembles the human type 2 DM phenotype. Therefore, more reliable results can be obtained concerning the clinical implications of the data. Additionally, fracture healing was assessed from biomechanical, radiological, and histopathological perspectives, ensuring that multiple methods were addressed within the same study. Moreover, although the effects of metformin and liraglutide on fracture healing have been explored in limited studies, comparing the two drugs in a single study and examining their combined effects is valuable.

This study has some limitations. The small sample size, due to ethical concerns, is a constraint. Because conventional X-ray radiological examinations are inadequate, more detailed methods like micro-CT should be used in future studies. The short follow-up period restricts the ability to assess long-term fracture healing, and studies with longer follow-up could better evaluate the later stages of remodeling. It might be helpful to evaluate blood samples taken during the euthanasia for some biochemical parameters and assess AGE accumulation and GLP-1R expression in bone tissue. The study was not examined at a mechanistic level; therefore, our findings should be considered hypothesis-generating. Metformin was given by gavage once daily, resulting in only a few hours of significant plasma concentrations each day, unlike the 24-hour profile seen in treated humans. Lastly, the study did not compare different dosages and application periods of the drugs used.

From a translational perspective, our findings could have clinical significance for managing diabetic patients with fractures. Current guidelines primarily focus on glycemic control when selecting antidiabetic treatments; however, our results suggest that medication selection may also impact bone healing. Notably, the combined use of metformin and liraglutide, both commonly prescribed for type 2 diabetes, may offer dual benefits by supporting metabolic regulation and promoting fracture repair. Although further clinical trials are needed, these findings highlight the potential to include bone health considerations in treatment strategies for diabetic fracture patients.

In conclusion, complications such as delayed fracture healing result in extended treatment durations, additional health issues, substantial increases in costs, and ultimately, profound impacts on patients’ quality of life. This study demonstrates that both metformin and liraglutide exhibit positive effects on fracture healing in a partially insulinopenic DM rat model. The combined administration of these two pharmaceuticals demonstrated that the adverse impact of DM on fracture healing can be broadly mitigated, resulting in outcomes comparable to those observed in the non-diabetic control group of rats. It is evident that these two medications, which are presently extensively utilized in the treatment of both pediatric, adolescent, and adult patients with type 2 DM, may facilitate fracture healing. Furthermore, combination therapies employing these agents possess significant potential for enhanced therapeutic effects. Future human-based studies are needed to support the data obtained from this study.

## Data Availability

The original contributions presented in the study are included in the article/**Supplementary Material**. Further inquiries can be directed to the corresponding author.
