# Supplementary material for: The effects of liraglutide and metformin treatment on fracture healing in partially insulinopenic diabetic rats
Source: Front Endocrinol (Lausanne). 2025 Oct 21;16:1703958. doi: 10.3389/fendo.2025.1703958 (PMC12582942; doi:10.3389/fendo.2025.1703958)
Supplement: Supplementary file 1 [file Table1.docx]

**Supplementary Tables**

**Supplementary Table 1:** Comparison of blood glucose levels between groups during the study period (Within-group post hoc p values).

|  | **Control** | **DM** | **Met** | **L** | **Met+L** |  |
| --- | --- | --- | --- | --- | --- | --- |
| **p_B-STZ_**  **p_B-1_**  **p_B-2_**  **p_B-3_**  **p_B-4_**  **p_B-5_**  **p_B-6_**  **p_STZ-1_**  **p_STZ-2_**  **p_STZ-3_**  **p_STZ-4_**  **p_STZ-5_**  **p_STZ-6_**  **p_1-2_**  **p_1-3_**  **p_1-4_**  **p_1-5_**  **p_1-6_**  **p_2-3_**  **p_2-4_**  **p_2-5_**  **p_2-6_**  **p_3-4_**  **p_3-5_**  **p_3-6_**  **p_4-5_**  **p_4-6_**  **p_5-6_** | 0.838  0.750  0.908  0.665  0.473  0.900  0.980  0.908  0.929  0.803  0.593  0.985  0.866  0.838  0.883  0.666  0.920  0.802  0.742  0.539  0.965  0.915  0.795  0.838  0.730  0.677  0.577  0.903 | **<0.001**  **<0.001**  **<0.001**  **<0.001**  **<0.001**  **<0.001**  **<0.001**  0.057  **0.014**  **0.025**  **0.002**  0.141  0.077  0.571  0.582  0.153  0.900  0.670  0.965  0.356  0.783  0.980  0.422  0.770  0.993  0.343  0.506  0.806 | **<0.001**  **<0.001**  **<0.001**  **<0.001**  **<0.001**  **<0.001**  **<0.001**  **<0.001**  **<0.001**  **<0.001**  **<0.001**  **<0.001**  **<0.001**  0.626  0.307  0.154  0.284  0.430  0.557  0.322  0.467  0.656  0.712  0.804  0.986  0.958  0.750  0.818 | **<0.001**  **<0.001**  **<0.001**  **<0.001**  **<0.001**  **<0.001**  **<0.001**  **<0.001**  **<0.001**  **<0.001**  **<0.001**  **<0.001**  **<0.001**  0.159  0.235  **0.039**  0.095  0.066  0.943  0.419  0.499  0.398  0.422  0.489  0.395  0.972  0.845  0.890 | **<0.001**  **<0.001**  **<0.001**  **<0.001**  **<0.001**  **<0.001**  **<0.001**  **<0.001**  **<0.001**  **<0.001**  **<0.001**  **<0.001**  **<0.001**  0.325  0.091  0.103  0.130  0.155  0.417  0.451  0.412  0.467  0.957  0.866  0.937  0.832  0.901  0.939 |  |

*STZ: 1 week after streptozotocin application, B: Baseline, 1 to 6: Weeks 1 to 6.*

*Within-group pairwise comparisons showed no significant changes across the study period in the control group (p>0.05 for all), all STZ-induced groups exhibited significant increases from baseline to post-STZ and subsequent weeks (p<0.001 for all), with no significant differences between weeks 1 to 6 (p>0.05 for all within-group comparisons of weeks 1 to 6)*

**Supplementary Table 2:** Comparison of weights between groups during the study period (Within-group post hoc p values).

|  | **Control** | | **DM** | | | **Met** | | **L** | | **Met+L** |  |
| --- | --- | --- | --- | --- | --- | --- | --- | --- | --- | --- | --- |
| **p_B-1_**  **p_B-2_**  **p_B-3_**  **p_B-4_**  **p_B-5_**  **p_B-6_**  **p_1-2_**  **p_1-3_**  **p_1-4_**  **p_1-5_**  **p_1-6_**  **p_2-3_**  **p_2-4_**  **p_2-5_**  **p_2-6_**  **p_3-4_**  **p_3-5_**  **p_3-6_**  **p_4-5_**  **p_4-6_**  **p_5-6_** | | **<0.001**  **<0.001**  **<0.001**  **0.045**  0.676  **0.001**  0.133  0.179  **0.002**  **<0.001**  **<0.001**  **0.007**  **<0.001**  **<0.001**  **<0.001**  0.117  **0.002**  **<0.001**  0.059  **<0.001**  **0.008** | | **<0.001**  **<0.001**  **<0.001**  **<0.001**  0.058  0.796  **0.039**  0.518  0.378  0.088  **0.001**  0.227  **0.007**  **0.002**  **<0.001**  0.164  **0.037**  **<0.001**  0.339  **0.013**  0.181 | **<0.001**  **<0.001**  **<0.001**  **0.003**  0.294  0.676  **0.019**  0.585  0.445  0.053  **0.001**  0.118  **0.004**  **<0.001**  **<0.001**  0.232  **0.026**  **<0.001**  0.208  **0.009**  0.231 | | **<0.001**  **<0.001**  **<0.001**  **0.022**  0.575  0.897  0.104  0.971  0.123  **0.014**  **0.002**  0.156  **0.003**  **<0.001**  **<0.001**  0.149  **0.019**  **0.003**  0.238  0.068  0.573 | | **<0.001**  **<0.001**  **<0.001**  **<0.001**  **<0.001**  **<0.001**  **0.001**  **0.019**  0.154  0.350  0.421  0.591  0.144  0.177  0.139  0.397  0.382  0.319  0.855  0.760  0.916 | |  |

*B: Baseline, 1 to 6: weeks 1 to six. Within-group pairwise comparisons showed complex patterns of significant and non-significant differences across groups and time points. Due to the extensive number of comparisons,*
